# The Topography of Poverty

**Published:** 2008-03-15

**Authors:** Christopher M G Buttery

**Affiliations:** Department of Epidemiology and Community Health, School of Medicine, Virginia Commonwealth University, Richmond, Virginia

## To the Editor:

"The Topography of Poverty in the United States: A Spatial Analysis Using County-Level Data From the Community Health Status Indicators Project" (1) is a fascinating article that describes how county-level poverty data were created into maps. Although appropriate for review by my students, the article raises the issue of how to explain such maps to elected officials. I have a hard enough time understanding the math, and I doubt whether one in a thousand elected officials could understand the maps or the data analysis if the mathematical formulae had to be explained. As a past local, regional, and state health director, I have had to elucidate many complex issues to elected officials to persuade them to implement policies that would improve the health of those for whom I was responsible. Is there some way to add transitional explanations to articles such as this one that would allow my students to use the maps (or similar maps) to work with policy makers during their internships to improve the health status of our communities?

**Read the author's reply**
